# Datasets for liquefaction case studies of gravelly soils during the 2008 Wenchuan earthquake

**DOI:** 10.1016/j.dib.2020.106308

**Published:** 2020-09-14

**Authors:** Yan-Guo Zhou, Peng Xia, Dao-Sheng Ling, Yun-Min Chen

**Affiliations:** MOE Key Laboratory of Soft Soils and Geoenvironmental Engineering, Institute of Geotechnical Engineering, Center for Hypergravity Experimental and Interdisciplinary Research, Zhejiang University, Hangzhou 310058, P. R. China

**Keywords:** The 2008 Wenchuan earthquake, Soil liquefaction, Gravelly soils, Shear wave velocity, Case histories

## Abstract

This Data in Brief article provides summarized information of the liquefaction case histories from the 12 May 2008 *M*_w_7.9 Wenchuan earthquake. According to the data processing procedures recommended in the related research article, all eighty-one case histories investigated by the present authors and seven other case histories from the literature are carefully compiled. All necessary information and the mean and standard deviation for some key parameters are given for these liquefaction case histories (i.e., site name, site location, site inclination angle, liquefaction manifestations, critical layer, soil sampling information, ground motion, total and effective stresses, shear-mass participation parameter, cyclic stress ratio (CSR) for *M*_w_=7.5, normalized shear wave velocity (*V*_s1_), data class and field testing time). These data may be used by colleagues to study the effect of gravel content on liquefaction behaviour of gravelly soils and develop the corresponding deterministic or probabilistic methods for liquefaction triggering evaluation.

## Specifications Table

**Table:** 

Subject	Geotechnical Engineering and Engineering Geology
Specific subject area	Soil liquefaction, Soil dynamics, Geotechnical earthquake engineering
Type of data	Table
How data were acquired	Site performance: field observations; Soil characteristics: field test pit, borehole sampling and laboratory tests; Site inclination angle: digital laser range finder; Ground motion parameters: USGS ShakeMap for the 2008 Wenchuan earthquake (URL: https://earthquake.usgs.gov/earthquakes/eventpage/usp000g650#shakemap) Shear wave velocity (*V*_s_): spectral analysis of surface waves (SASW) tests or down-hole tests; Total and effective stresses, shear-mass participation parameter, cyclic stress ratio (CSR): calculated according to the data processing procedures introduced in the related research article
Data format	Data are in raw format and also have been analyzed. A Microsoft Excel spreadsheet file of raw data is available for public access.
Parameters for data collection	Site name, site location, site inclination angle, liquefaction manifestation, critical layer depth, soil sampling information, PGA, total and effective overburden stresses, shear-mass participation parameter, CSR for *M*_w_=7.5, measured and corrected shear wave velocity, data class, field testing time.
Description of data collection	The data was collected by site investigations right after the 2008 Wenchuan earthquake and some laboratory tests, including the field measurement of site inclination angle, the observation of liquefaction manifestations, performances of infrastructures built upon the site, the field borehole sampling and shear wave velocity measurements, as well as the PGA estimated according to the USGS ShakeMap.
Data source location	Zhejiang University, Zijingang Campus, Hangzhou 310058, Zhejiang Province, PR China
Data accessibility	Raw data could be retrieved from the homepage of the corresponding author, and please visit the following link and find the attachment "Raw data DIB-S-20-01747.xlsx" on the webpage: https://person.zju.edu.cn/en/zhouyanguo#757551 Or directly copy the following URL to a browser (Google Chrome is recommended) to download the spreadsheet file: https://person.zju.edu.cn/person/wescms/sys/filebrowser/file.php?cmd=download&id=835988
Related research article	Yan-Guo Zhou, Peng Xia, Dao-Sheng Ling, Yun-Min Chen, 2020. Liquefaction case studies of gravelly soils during the 2008 Wenchuan earthquake. Engineering Geology, Vol. 274C, 105691. https://doi.org/10.1016/j.enggeo.2020.105691

## Value of the Data

• These data are useful to enrich the global database of field liquefaction case histories with emphasis on gravelly soils and high seismic intensity.
• These data could be of benefit to other researchers to develop new liquefaction triggering boundary curves and the corresponding liquefaction evaluation method for gravelly soils.
• These data give an insight into the gravel content effect on soil stiffness and liquefaction resistance of sand-gravel mixtures, which urges more detailed element tests to understand the liquefaction behaviors of gravelly soils.
• These data encourage researchers to carry out further physical modelling and numerical analysis to reveal the dynamic response and liquefaction hazard of gravelly soil deposits.

## 1 Data Description

**Fig. 1:**
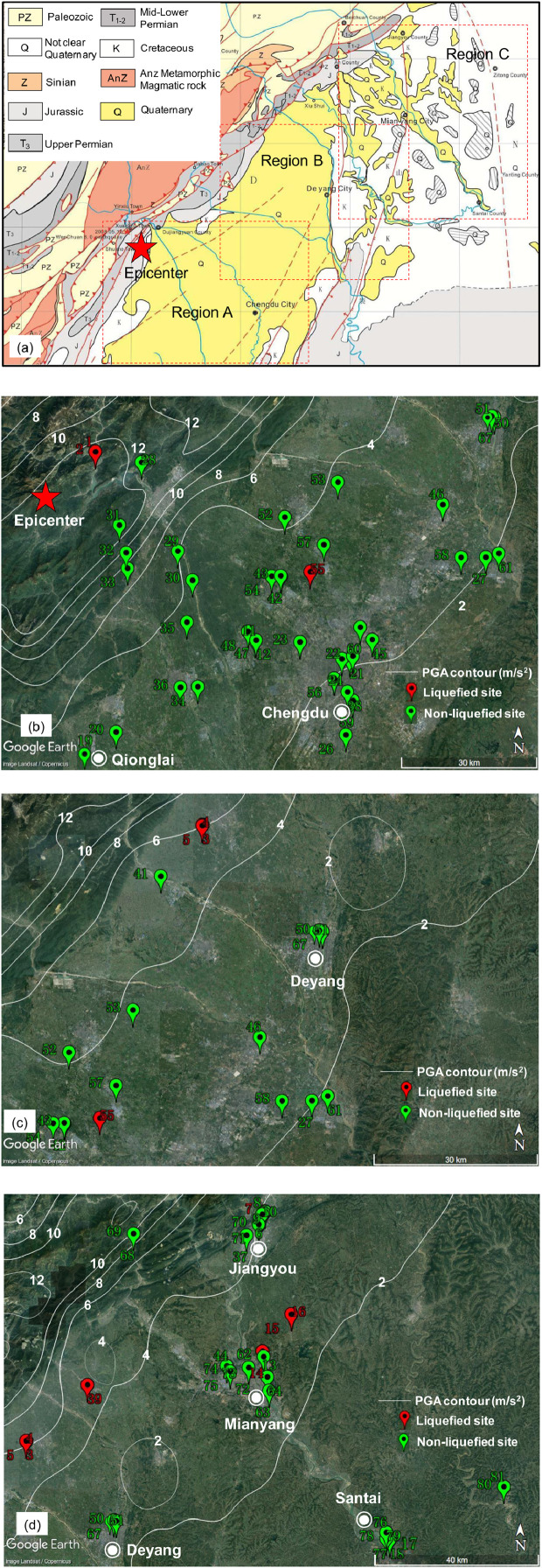
Map of the investigated sites: (a) geologic settings (modified from Zhao et al.[3]); (b) cases in Region A; (c) cases in Region B; (d) cases in Region C.

All 81 case histories compiled by the authors and 7 case histories re-compiled from Cao (2010) [2] are summarized in Table 1, which is given at the end of this article. The investigated sites spread throughout an area of 200 km × 180 km and are mainly distributed in three regions, as shown in Fig. 1(a). Detailed locations of these investigated sites in the three regions are marked in Fig. 1(b)–(d), and most of these cases spread in Chengdu plain area and a few others are in mountain valleys. Note that the case number of each site shown in Fig. 1(b)–(d) are consistent with the spreadsheet file of raw data provided in the above Specifications Table. In this compiled database, some basic information including the site name, location coordinates, site inclination angle, liquefaction information, ground water table, soil classification, permeability and gravel content (GC) are obtained by field investigation and testing, borehole sampling and laboratory tests. The other parameters like the depth of critical layer, peak ground acceleration, total and effective overburden stresses, shear-mass participation parameter, cyclic stress ratio, overburden stress-corrected shear wave velocity are determined or calculated according to the data processing procedures introduced in the related research article [1].

**Table 1:** Summary of (CSR, *V*_s1_) field case hisotries during the 2008 Wenchuan earthquake.

Case No.	Site name	Location coordinates	Lique- fied?	GWT (m)	Critical depth range (m)	Soil classification	Permeability (cm/s)	GC (%)	Inclination angle (°)	PGA (g)	*σ*_v_ (kPa)	*σ′*_v_ (kPa)	*r*_d_	CSR_7.5_	Overburden stress corrected *V*_s1_ (m/s)	Data class	Field testing time
1	Minjiang flood plain ZK3	31.045°N,103.477°E	Yes	2.50	2.50–4.31	gravelly sand	0.032	40.0	4.00	1.457 ± 0.182	63.3 ± 7.7	54.4 ± 6.2	0.994 ± 0.056	1.218 ± 0.262	320.2 ± 19.0	B	2008.11
2	Minjiang flood plain ZK5	31.045°N, 103.477°E	Yes	1.20	1.20–3.20	gravelly sand	0.032	40.0	4.00	1.457 ± 0.182	40.1 ± 6.9	30.3 ± 4.9	0.997 ± 0.039	1.389 ± 0.371	308.1 ± 18.7	B	2008.11
3	Mianzhu Banqiao School XK5	31.299°N, 104.165°E	Yes	2.47	2.47–5.00	gravelly sand	0.020	25.0	0.00	0.577 ± 0.105	70.0 ± 9.8	57.6 ± 7.1	0.996 ± 0.061	0.504 ± 0.134	202.1 ± 23.9	B	2008.11
4	Mianzhu Banqiao School XK15	31.299°N, 104.165°E	Yes	3.46	3.46–5.00	gravelly sand	0.020	35.0	0.00	0.577 ± 0.105	80.2 ± 8.5	72.6 ± 7.6	0.995 ± 0.068	0.458 ± 0.111	218.0 ± 18.3	B	2008.11
5	Mianzhu Banqiao School XK26	31.298°N, 104.165°E	Yes	2.60	2.60–6.00	gravelly sand	0.020	35.0	0.00	0.577 ± 0.105	81.6 ± 12.8	65.0 ± 8.6	0.994 ± 0.069	0.521 ± 0.147	257.9 ± 42.0	B	2008.11
6	Jiangyou thermal power plant ZK3	31.805°N, 104.770°E	No	3.90	3.90–6.00	sandy gravel	0.058	54.0	0.00	0.361 ± 0.089	95.0 ± 10.5	84.7 ± 8.9	0.994 ± 0.078	0.291 ± 0.087	283.6 ± 41.9	B	2008.12
7	Jiangyou thermal power plant ZK5	31.806°N,104.769°E	Yes	3.90	3.90–4.90	sand	0.058	10.0	0.00	0.361 ± 0.089	83.8 ± 8.2	78.9 ± 8.0	0.995 ± 0.070	0.276 ± 0.080	201.0 ± 5.0	B	2008.12
8	Jiangyou thermal power plant Site1	31.802°N,104.766°E	No	3.20	4.80–8.00	sandy gravel	0.058	50.0	0.00	0.361 ± 0.089	123.9 ± 14.0	92.5 ± 10.7	0.990 ± 0.096	0.346 ± 0.107	290.3 ± 32.8	B	2008.12
9	Jiangyou thermal power plant Site2	31.802°N,104.764°E	No	2.70	3.50–4.60	gravelly sand	0.058	40.0	0.00	0.361 ± 0.089	76.5 ± 6.7	63.3 ± 6.4	0.996 ± 0.065	0.314 ± 0.090	212.3 ± 1.0	B	2008.12
10	Jiangyou thermal power plant Site3	31.799°N,104.763°E	No	3.20	3.20–5.00	sandy gravel	0.058	56.0	0.00	0.361 ± 0.089	76.5 ± 8.5	67.7 ± 7.3	0.996 ± 0.066	0.294 ± 0.087	303.2 ± 10.8	B	2008.12
11	Wenjiang construction site ZK140	30.678°N,103.854°E	No	2.10	2.10– 4.00	sandy gravel	0.023	54.0	0.00	0.263 ± 0.077	59.3 ± 8.3	48.5 ± 6.2	0.998 ± 0.051	0.227 ± 0.079	311.8 ± 52.3	C	2008.12
12	Wenjiang construction site ZK142	30.679°N,103.856°E	No	2.50	2.50–4.70	sandy gravel	0.023	70.0	0.00	0.263 ± 0.077	67.1 ± 8.7	56.3 ± 6.7	0.997 ± 0.059	0.226 ± 0.078	385.7 ± 21.3	B	2008.12
13	Mianyang Fengtai ZK2	31.501°N,104.766°E	No	5.00	6.60–7.40	gravelly sand	–	25.0	2.70	0.228 ± 0.070	137.2 ± 10.8	117.6 ± 10.8	0.957 ± 0.104	0.184 ± 0.064	227.2 ± 10.3	B	2008.11
14	Mianyang Fengtai ZK12	31.501°N,104.767°E	Yes	3.00	3.00–5.90	sand	–	0.0	2.70	0.228 ± 0.070	84.1 ± 11.4	69.9 ± 8.3	0.983 ± 0.070	0.195 ± 0.071	178.9 ± 10.2	B	2008.11
15	Mianyang Jiezi Element School Site1	31.582°N,104.840°E	Yes	2.50	2.50–4.00	sand	–	0.0	0.00	0.240 ± 0.078	60.6 ± 6.9	53.2 ± 6.0	0.998 ± 0.054	0.197 ± 0.072	167.8 ± 9.6	C	2008.11
16	Mianyang Jiezi Element School Site2	31.582°N,104.840°E	Yes	3.00	3.00–5.00	sand	–	0.0	0.00	0.240 ± 0.078	75.5 ± 8.9	65.7 ± 7.2	0.996 ± 0.064	0.198 ± 0.073	173.7 ± 1.7	C	2008.11
17	Santai construction site Site48	31.091°N,105.096°E	No	3.00	3.00–5.00	sand	–	2.5	1.10	0.126 ± 0.051	74.5 ± 8.8	64.7 ± 7.1	0.997 ± 0.064	0.105 ± 0.046	200.6 ± 10.0	C	2008.11
18	Santai construction site Site49	31.092°N,105.096°E	No	3.30	3.30–4.00	sand	–	8.0	1.10	0.126 ± 0.051	67.6 ± 6.5	64.2 ± 6.7	0.997 ± 0.060	0.096 ± 0.041	212.5 ± 4.3	C	2008.11
19	Qionglai factory site	30.398°N,103.482°E	No	1.50	2.00–3.00	sand	0.023	20.0	0.00	0.257 ± 0.073	45.2 ± 4.5	35.4 ± 4.4	0.998 ± 0.043	0.237 ± 0.078	204.3 ± 4.1	B	2008.11
20	Dayi Sancha Middle School	30.492°N,103.538°E	No	3.20	3.20–6.00	sand	0.015	5.0	0.00	0.260 ± 0.072	86.4 ± 11.3	72.7 ± 8.4	0.995 ± 0.072	0.222 ± 0.074	186.2 ± 11.4	B	2008.11
21	Chengdu Taishengnanlu	30.664°N,104.072°E	No	4.95	6.70–10.20	sand	0.021	10.0	0.00	0.221 ± 0.067	156.1 ± 15.8	121.8 ± 12.7	0.844 ± 0.121	0.173 ± 0.063	180.5 ± 3.7	C	2007.11
22	Chengdu Jinjiangqu	30.651°N,104.079°E	No	6.45	6.45–7.70	sand	0.021	15.0	0.00	0.178 ± 0.059	129.5 ± 12.5	123.4 ± 12.3	0.961 ± 0.104	0.130 ± 0.049	212.7 ± 29.8	C	2007.04
23	Chengdu Guanghuajinyuan	30.677°N,103.957°E	No	4.50	4.50–6.80	sand	0.023	10.0	0.00	0.245 ± 0.072	106.2 ± 11.4	95.0 ± 9.8	0.985 ± 0.086	0.195 ± 0.067	222.8 ± 0.0	B	2011.01
24	Chengdu Wujing Hospital	30.645°N,104.054°E	No	3.00	3.80–8.00	sand	0.023	20.0	0.00	0.188 ± 0.060	110.4 ± 15.4	81.9 ± 10.5	0.988 ± 0.090	0.181 ± 0.069	234.5 ± 8.3	C	2013.01
25	Chengdu Zhiminlu	30.646°N,104.766°E	No	6.00	6.00–8.30	sandy gravel	0.021	51.0	0.00	0.126 ± 0.051	129.3 ± 13.2	118.1 ± 11.8	0.989 ± 0.105	0.099 ± 0.044	240.9 ± 17.9	C	2005.07
26	Chengdu Huayang	30.495°N,104.066°E	No	3.90	4.80–9.00	sand	0.023	24.0	0.00	0.135 ± 0.048	130.9 ± 16.3	101.5 ± 11.7	0.990 ± 0.102	0.125 ± 0.051	251.6 ± 2.5	C	2009.11
27	Chengdu Jintangzhaozheng	30.850°N,104.382°E	No	1.00	3.40–4.80	sand	0.081	12.0	0.00	0.197 ± 0.068	77.2 ± 7.6	46.8 ± 7.1	0.996 ± 0.066	0.235 ± 0.092	222.2 ± 0.9	C	2013.07
28	Dujiangyan Zipingpu School	31.026°N,103.584°E	No	2.40	3.30–4.80	gravelly sand	-	32.0	5.60	0.725 ± 0.123	75.0 ± 7.2	58.8 ± 6.4	0.994 ± 0.065	0.664 ± 0.154	282.3 ± 1.9	B	2008.09
29	Dujiangyan Xudu Element School	30.852°N,103.672°E	No	2.20	2.20–2.80	sandy gravel	0.023	50.0	0.00	0.408 ± 0.092	45.7 ± 4.5	42.7 ± 4.8	0.998 ± 0.043	0.315 ± 0.085	245.7 ± 4.9	B	2008.10
30	Dujiangyan Yanjiang Element School	30.795°N,103.707°E	No	2.50	2.50–4.50	sandy gravel	0.035	60.0	0.00	0.392 ± 0.095	64.6 ± 8.7	54.8 ± 6.4	0.997 ± 0.057	0.333 ± 0.102	369.1 ± 29.5	B	2008.09
31	Dujiangyan Shaping School	30.901°N,103.536°E	No	4.50	4.50–7.40	sandy gravel	0.023	55.0	3.55	1.246 ± 0.179	111.3 ± 13.4	97.1 ± 10.4	0.984 ± 0.090	1.016 ± 0.237	277.0 ± 33.2	B	2008.10
32	Dujiangyan Lianghe School	30.847°N,103.553°E	No	3.90	4.40–5.10	sand	0.023	15.0	6.45	0.725 ± 0.136	89.8 ± 7.8	81.5 ± 8.0	0.993 ± 0.074	0.573 ± 0.138	202.1 ± 4.0	B	2008.11
33	Congzhou Jiezi Hospital	30.816°N,103.557°E	No	6.00	6.00–7.20	sandy gravel	0.023	65.0	0.69	0.647 ± 0.129	128.0 ± 12.4	122.1 ± 12.0	0.986 ± 0.098	0.483 ± 0.126	362.8 ± 18.1	B	2008.10
34	Chongzhou Dahua Hospital	30.585°N,103.724°E	No	1.85	2.70–4.70	sandy gravel	0.023	55.0	0.00	0.275 ± 0.079	70.1 ± 8.2	52.0 ± 6.5	0.997 ± 0.060	0.267 ± 0.091	283.9 ± 22.7	B	2008.10
35	Chongzhou Liaojia Hospital	30.712°N,103.696°E	No	2.00	2.20–3.00	gravelly sand	0.023	35.0	0.57	0.341 ± 0.088	46.4 ± 4.5	40.5 ± 4.7	0.998 ± 0.045	0.282 ± 0.085	277.5 ± 14.2	B	2008.10
36	Chongzhou Jixian Hospital	30.583°N,103.684°E	No	2.05	3.10–4.50	gravelly sand	0.023	28.0	0.46	0.284 ± 0.079	66.4 ± 6.3	49.3 ± 5.7	0.997 ± 0.062	0.276 ± 0.089	277.0 ± 22.2	B	2008.10
37	Jiangyou Longxigu	31.753°N,104.723°E	No	4.72	4.72– 6.20	gravelly sand	-	45.0	0.00	0.428 ± 0.086	105.3 ± 10.4	98.1 ± 9.9	0.992 ± 0.084	0.330 ± 0.085	258.2 ± 20.7	B	2008.07
38	Chengdu Middle Court	30.580°N,104.068°E	No	8.00	10.40–14.70	sand	0.023	10.0	0.00	0.133 ± 0.045	237.4 ± 21.9	192.8 ± 18.8	0.806 ± 0.170	0.096 ± 0.040	213.3 ± 4.2	C	2006.09
39	Mianzhu Mianyuan School	31.423°N,104.320°E	Yes	2.00	2.00–3.60	gravelly sand	0.058	28.0	0.00	0.443 ± 0.101	51.3 ± 6.5	43.5 ± 5.3	0.998 ± 0.048	0.377 ± 0.110	227.0 ± 23.2	B	2008.09
40	Wenchuan Mianshi Cherry	31.399°N,103.521°E	No	3.00	3.00–4.30	sandy gravel	0.116	60.0	-	0.754 ± 0.135	76.4 ± 7.9	70.1 ± 7.4	0.995 ± 0.060	0.592 ± 0.141	308.9 ± 5.0	B	2010.02
41	Shifang Lingjie Hospital	31.214°N,104.087°E	No	7.00	7.00–9.00	sandy gravel	0.023	57.5	0.69	0.449 ± 0.098	154.0 ± 15.2	144.2 ± 14.2	0.980 ± 0.116	0.340 ± 0.097	290.9 ± 23.3	B	2009.04
42	Pixian Huayutianfu ZK26	30.806°N,103.911°E	No	5.00	5.00–6.30	sandy gravel	0.025	63.0	0.52	0.291 ± 0.081	108.0 ± 10.6	101.6 ± 10.1	0.993 ± 0.086	0.222 ± 0.072	354.9 ± 28.4	B	2014.03
43	Pixian Huayutianfu ZK53	30.807°N,103.910°E	No	5.00	5.40–8.40	sandy gravel	0.025	50.0	0.52	0.291 ± 0.081	126.6 ± 14.0	108.0 ± 11.2	0.988 ± 0.102	0.244 ± 0.081	237.7 ± 33.3	B	2014.03
44	Mianyang Fanhua Plaza	31.468°N,104.676°E	No	5.90	5.90– 6.80	sandy gravel	0.053	52.0	1.43	0.322 ± 0.088	130.9 ± 12.6	126.5 ± 12.6	0.990 ± 0.095	0.239 ± 0.077	250.4 ± 5.0	B	2013.11
45	Chengdu Erxianqiao	30.684°N,104.123°E	No	7.20	7.20–9.30	gravelly sand	0.023	25.0	0.00	0.220 ± 0.068	175.8 ± 17.3	165.5 ± 16.2	0.983 ± 0.119	0.166 ± 0.060	217.5 ± 2.3	C	2013.10
46	Guanhan Hanzhuang	30.953°N,104.281°E	No	4.90	6.60––8.30	sand	0.058	5.0	0.46	0.235 ± 0.073	139.4 ± 11.6	114.4 ± 11.0	0.970 ± 0.109	0.201 ± 0.071	205.8 ± 18.4	C	2010.11
47	Wenjiang Campus,Chengdu Medicine Univ. ZK65	30.696°N,103.837°E	No	2.50	2.70–3.30	sandy gravel	0.023	55.0	0.69	0.278 ± 0.079	54.6 ± 5.0	49.7 ± 5.5	0.998 ± 0.050	0.220 ± 0.071	310.1 ± 6.2	B	2012.10
48	Wenjiang Campus,Chengdu Medicine Univ. ZK134	30.696°N, 103.837°E	No	2.50	2.50–4.60	sandy gravel	0.023	55.0	0.69	0.278 ± 0.079	64.3 ± 8.1	54.0 ± 6.4	0.997 ± 0.058	0.239 ± 0.081	340.9 ± 27.3	B	2012.10
49	Jiangyou Shuangma	31.991°N,105.040°E	No	3.30	3.50–5.80	sandy gravel	0.023	58.0	1.32	0.369 ± 0.091	89.0 ± 10.2	75.8 ± 8.2	0.995 ± 0.073	0.311 ± 0.094	317.2 ± 25.4	B	2010.06
50	Deyang Yuanyou	31.127°N,104.398°E	No	11.00	11.00–15.00	sand	0.046	13.0	0.00	0.220 ± 0.069	232.6 ± 23.5	213.0 ± 20.9	0.785 ± 0.175	0.136 ± 0.056	186.0 ± 0.1	C	2011.01
51	Deyang Fukang	31.134°N,104.382°E	No	9.90	9.90–12.70	gravelly sand	0.069	40.0	0.00	0.227 ± 0.069	216.5 ± 21.2	202.8 ± 19.8	0.894 ± 0.156	0.157 ± 0.059	235.9 ± 18.9	C	2011.05
52	Pengzhou Qingbaijiang bridge	30.923°N,103.917°E	No	0.50	0.60– 1.20	gravelly sand	0.025	33.0	0.04	0.389 ± 0.090	17.8 ± 2.3	13.9 ± 3.5	0.999 ± 0.018	0.361 ± 0.130	252.0 ± 5.0	C	2004.03
53	Pengzhou Jiuchimao	30.994°N,104.038°E	No	2.70	2.70–3.80	sandy gravel	-	50.0	0.00	0.237 ± 0.073	64.2 ± 6.6	58.8 ± 6.3	0.998 ± 0.054	0.186 ± 0.064	255.3 ± 20.4	B	2009.08
54	Pixian south	30.806°N,103.889°E	No	2.10	2.10–4.90	sandy gravel	0.041	52.0	0.00	0.302 ± 0.094	66.3 ± 10.5	52.5 ± 7.1	0.997 ± 0.057	0.275 ± 0.104	335.7 ± 40.3	C	2011.07
55	Pixian Tuanjiezheng	30.816°N,103.978°E	Yes	1.80	1.90–3.10	sand	0.022	0.0	0.00	0.314 ± 0.082	47.8 ± 5.3	40.9 ± 5.2	0.998 ± 0.043	0.265 ± 0.083	173.8 ± 23.1	B	2008.12
56	Chengdu Shenxianshu	30.606°N,104.037°E	No	4.90	5.50–6.80	sand	0.023	5.0	0.00	0.166 ± 0.054	118.4 ± 10.6	106.2 ± 10.4	0.992 ± 0.093	0.133 ± 0.048	189.0 ± 9.5	C	2010.01
57	Chengdu Xinfanzheng	30.870°N,104.008°E	No	4.13	4.13–7.40	gravelly sand	0.023	28.0	0.00	0.282 ± 0.080	111.3 ± 14.0	95.3 ± 10.5	0.992 ± 0.088	0.236 ± 0.081	209.0 ± 35.6	B	2008.07
58	Chengdu Tiantian	30.849°N,104.325°E	No	4.10	4.10–5.15	sandy gravel	0.023	50.0	0.00	0.206 ± 0.068	87.2 ± 8.5	82.1 ± 8.6	0.996 ± 0.073	0.158 ± 0.058	269.0 ± 13.5	C	2010.11
59	Chengdu Nanbu center	30.563°N,104.081°E	No	6.00	6.00–6.60	gravelly sand	0.023	30.0	0.00	0.116 ± 0.042	117.0 ± 11.3	114.1 ± 11.6	0.992 ± 0.095	0.086 ± 0.034	263.3 ± 5.3	C	2005.01
60	Chengdu Taiye plaza	30.708°N,104.095°E	No	5.00	5.00–6.50	gravelly sand	0.021	45.0	0.00	0.228 ± 0.068	109.2 ± 10.7	101.9 ± 10.3	0.993 ± 0.088	0.175 ± 0.060	285.4 ± 14.3	B	2012.05
61	Chengdu Jintanghexie	30.858°N,104.412°E	No	2.70	3.00–5.00	gravelly sand	0.020	30.0	0.00	0.194 ± 0.067	76.9 ± 8.7	64.1 ± 7.2	0.997 ± 0.064	0.168 ± 0.065	264.9 ± 7.5	C	2008.05
62	Mianyang Fuleyaju	31.489°N,104.770°E	No	4.51	5.00–8.00	sandy gravel	0.035	52.5	0.00	0.321 ± 0.085	123.1 ± 13.5	103.6 ± 10.8	0.973 ± 0.097	0.269 ± 0.086	268.2 ± 40.2	B	2010.08
63	Mianyang Fulin	31.415°N,104.785°E	No	3.60	3.60–5.10	gravelly sand	0.159	35.0	0.00	0.278 ± 0.077	86.4 ± 8.8	79.1 ± 8.3	0.996 ± 0.069	0.219 ± 0.070	246.4 ± 12.3	B	2009.05
64	Mianyang Sanjiang	31.445°N,104.780°E	No	5.40	5.40–7.90	sand	0.150	0.0	0.75	0.306 ± 0.083	129.3 ± 13.5	117.1 ± 11.9	0.961 ± 0.099	0.235 ± 0.076	170.7 ± 20.5	B	2009.12
65	Wenchuan Miansi	31.360°N,103.497°E	No	4.70	4.70–6.50	sandy gravel	0.035	80.0	0.86	0.683 ± 0.135	112.2 ± 11.4	103.4 ± 10.4	0.990 ± 0.086	0.530 ± 0.137	418.9 ± 20.9	B	2010.06
66	Wenchuan Weizhouzhen	31.457°N,103.561°E	Yes	5.65	5.65– 9.90	gravelly sand	0.058	40.0	5.71	0.747 ± 0.130	141.3 ± 16.9	120.4 ± 12.8	0.977 ± 0.113	0.619 ± 0.163	277.7 ± 55.5	B	2009.03
67	Deyang Changjiangxilu	31.129°N,104.392°E	No	8.50	8.50–14.30	gravelly sand	–	27.5	1.60	0.223 ± 0.069	214.5 ± 25.6	186.1 ± 19.6	0.850 ± 0.157	0.158 ± 0.062	225.4 ± 0.8	C	2014.02
68	Beichuan Xinfeng cement ZK48	31.754°N,104.433°E	No	2.00	2.80– 3.90	sandy gravel	–	53.0	1.15	0.678 ± 0.128	65.3 ± 6.0	52.1 ± 5.7	0.996 ± 0.055	0.612 ± 0.148	329.9 ± 3.3	B	2008.08
69	Beichuan Xinfeng cement ZK76	31.754°N,104.433°E	No	2.00	11.60–13.90	gravelly sand	–	35.0	1.15	0.678 ± 0.128	281.4 ± 25.7	176.0 ± 24.9	0.911 ± 0.172	0.714 ± 0.225	222.4 ± 26.7	B	2008.08
70	Jiangyou Lanwan ZK5	31.777°N,104.754°E	No	2.00	6.00–7.40	sandy gravel	–	55.0	0.34	0.369 ± 0.086	135.2 ± 11.6	89.2 ± 11.0	0.988 ± 0.100	0.399 ± 0.118	312.0 ± 25.0	B	2007.11
71	Jiangyou Lanwan ZK17	31.777°N,104.754°E	No	2.00	2.40–5.40	sandy gravel	–	70.0	0.34	0.369 ± 0.086	79.7 ± 12.6	61.1 ± 8.6	0.996 ± 0.063	0.346 ± 0.111	441.7 ± 66.3	B	2007.11
72	Mianyang Jinqiaoyinzuo ZK14	31.465°N,104.732°E	No	3.00	4.30–6.50	gravelly sand	–	42.5	0.00	0.326 ± 0.084	109.2 ± 11.2	85.7 ± 9.1	0.993 ± 0.083	0.298 ± 0.092	257.9 ± 5.0	B	2007.10
73	Mianyang Jinqiaoyinzuo ZK21	31.465°N,104.732°E	No	3.00	3.00–4.40	gravelly sand	–	44.0	0.00	0.327 ± 0.085	77.4 ± 8.2	70.6 ± 7.4	0.997 ± 0.060	0.259 ± 0.079	281.6 ± 8.3	B	2007.10
74	Mianyang Lingjiangdijing ZK3	31.456°N,104.685°E	No	1.50	2.00– 3.00	sand	–	5.0	–	0.327 ± 0.085	43.9 ± 4.4	34.1 ± 4.2	0.998 ± 0.043	0.304 ± 0.094	207.2 ± 4.1	B	2006.08
75	Mianyang Lingjiangdijing ZK15	31.456°N,104.685°E	No	1.50	2.00– 3.00	sand	–	8.0	–	0.327 ± 0.085	44.6 ± 4.5	34.8 ± 4.3	0.998 ± 0.043	0.303 ± 0.093	208.0 ± 4.2	B	2006.08
76	Santai Guixidijing ZK17	31.108°N,105.087°E	No	8.00	8.00–11.90	sandy gravel	–	50.0	–	0.121 ± 0.053	195.5 ± 21.3	176.3 ± 17.8	0.965 ± 0.140	0.093 ± 0.045	238.4 ± 10.2	C	2008.09
77	Santai Guixidijing ZK40	31.108°N,105.087°E	No	8.00	8.00–10.30	sandy gravel	–	55.0	–	0.121 ± 0.053	183.6 ± 18.1	172.4 ± 16.9	0.980 ± 0.130	0.091 ± 0.044	253.3 ± 30.4	C	2008.09
78	Santai Zizhou ZK2	31.095°N,105.087°E	No	9.00	9.00–11.90	gravelly sand	–	40.0	–	0.121 ± 0.050	204.5 ± 20.5	190.2 ± 18.6	0.895 ± 0.146	0.084 ± 0.039	220.7 ± 13.2	C	2008.08
79	Santai Zizhou ZK20	31.095°N,105.087°E	No	9.00	9.00–12.10	gravelly sand	–	37.0	–	0.121 ± 0.050	202.0 ± 20.5	186.8 ± 18.3	0.848 ± 0.147	0.080 ± 0.038	224.0 ± 16.7	C	2008.08
80	Yanting Zijiangxincheng ZK14	31.209°N,105.387°E	No	6.90	6.90–10.60	sand	–	15.0	–	0.109 ± 0.047	167.5 ± 18.2	149.4 ± 15.2	0.916 ± 0.125	0.081 ± 0.038	228.0 ± 34.2	C	2008.06
81	Yanting Zijiangxincheng ZK63	31.209°N,105.387°E	No	5.00	5.00–6.30	sand	–	5.0	–	0.109 ± 0.047	109.4 ± 10.6	103.1 ± 10.4	0.994 ± 0.086	0.083 ± 0.038	208.7 ± 10.4	C	2008.06
Cases recompiled from Cao (2010)																	
C1	Deyang Anping Village	31.320°E, 104.411°N	Yes	1.80	2.80–5.00	sandy gravel	–	60.0	–	0.170 ± 0.055	72.5 ± 8.7	51.9 ± 6.7	0.997 ± 0.063	0.171 ± 0.063	228.2 ± 26.8	C	2008.08
C2	Jiangyou Railway Station	31.794°E, 104.782°N	Yes	2.40	2.40–6.00	sandy gravel	–	62.0	–	0.354 ± 0.094	78.6 ± 12.9	61.0 ± 8.4	0.996 ± 0.067	0.328 ± 0.114	264.5 ± 13.7	B	2008.08
C3	Mianzhu Xiangliu Village	31.433°E, 104.241°N	Yes	3.40	3.40–6.00	sandy gravel	–	50.0	–	0.687 ± 0.121	89.1 ± 11.1	76.4 ± 8.5	0.989 ± 0.074	0.573 ± 0.146	257.9 ± 9.7	B	2008.08
C4	Mianzhu Anren Village	31.424°E, 104.208°N	Yes	4.00	4.80–7.00	sandy gravel	–	65.0	–	0.706 ± 0.118	113.6 ± 11.3	95.0 ± 9.6	0.988 ± 0.090	0.603 ± 0.143	277.4 ± 18.4	B	2008.08
C5	Deyang Dasheng Village	31.279°E, 104.236°N	No	4.50	5.50–8.00	sandy gravel	–	50.0	–	0.465 ± 0.094	131.8 ± 13.0	109.8 ± 11.0	0.987 ± 0.100	0.399 ± 0.106	246.2 ± 2.4	B	2008.08
C6	Mianzhu Linfa Village	31.432°E, 104.190°N	No	4.30	4.30–7.80	sandy gravel	–	70.0	–	0.785 ± 0.125	116.7 ± 14.9	99.6 ± 11.1	0.987 ± 0.091	0.657 ± 0.164	380.4 ± 9.7	B	2008.09
C7	Mianzhu Changlin Village	31.414°E, 104.152°N	No	4.00	4.00–6.00	sandy gravel	–	50.0	–	0.829 ± 0.120	95.1 ± 10.4	85.3 ± 8.8	0.991 ± 0.078	0.662 ± 0.148	247.7 ± 25.4	B	2008.09

## 2 Experimental Design, Materials and Methods

**Fig. 2:**
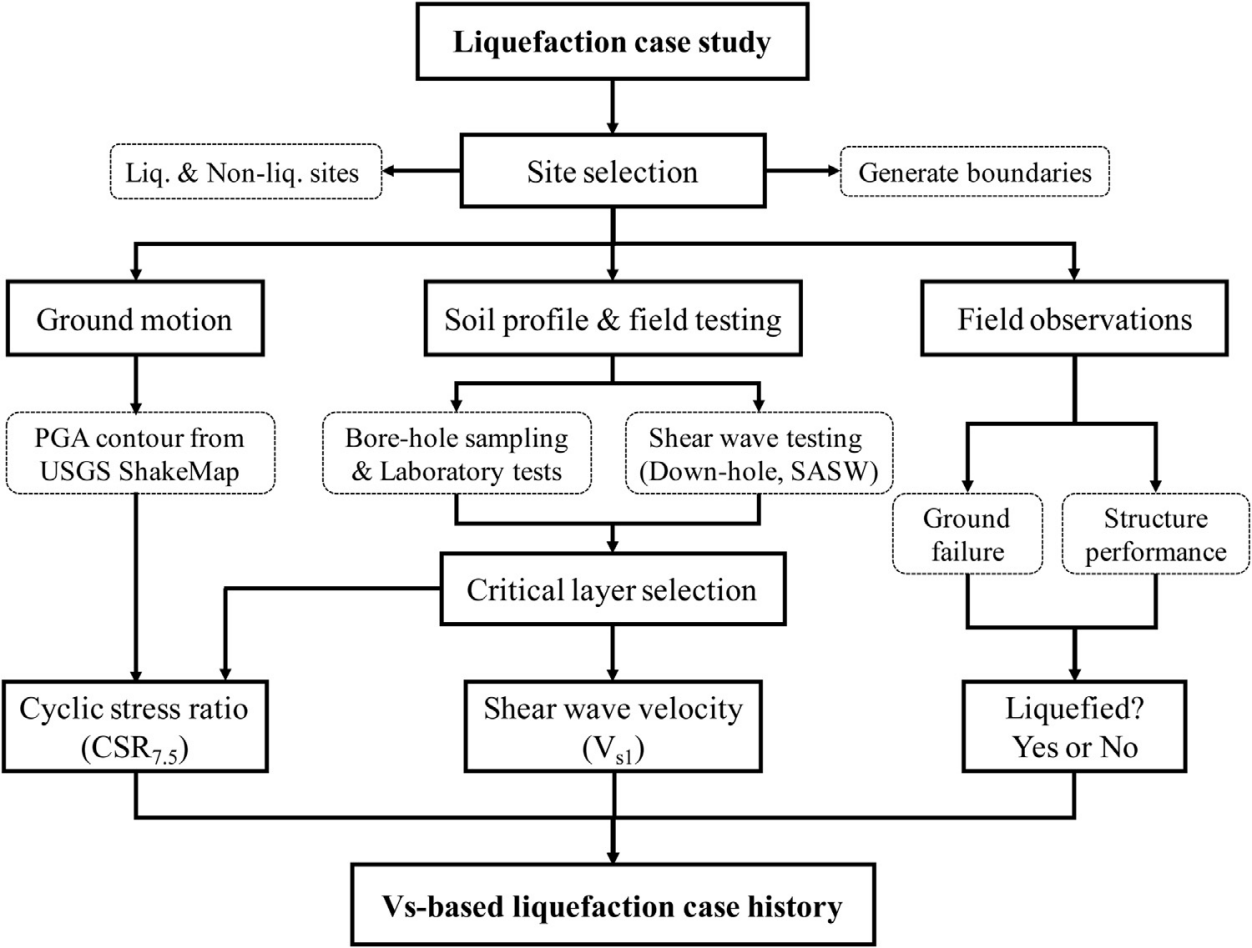
The procedure of *V*_s_-based liquefaction case study

**Fig. 3:**
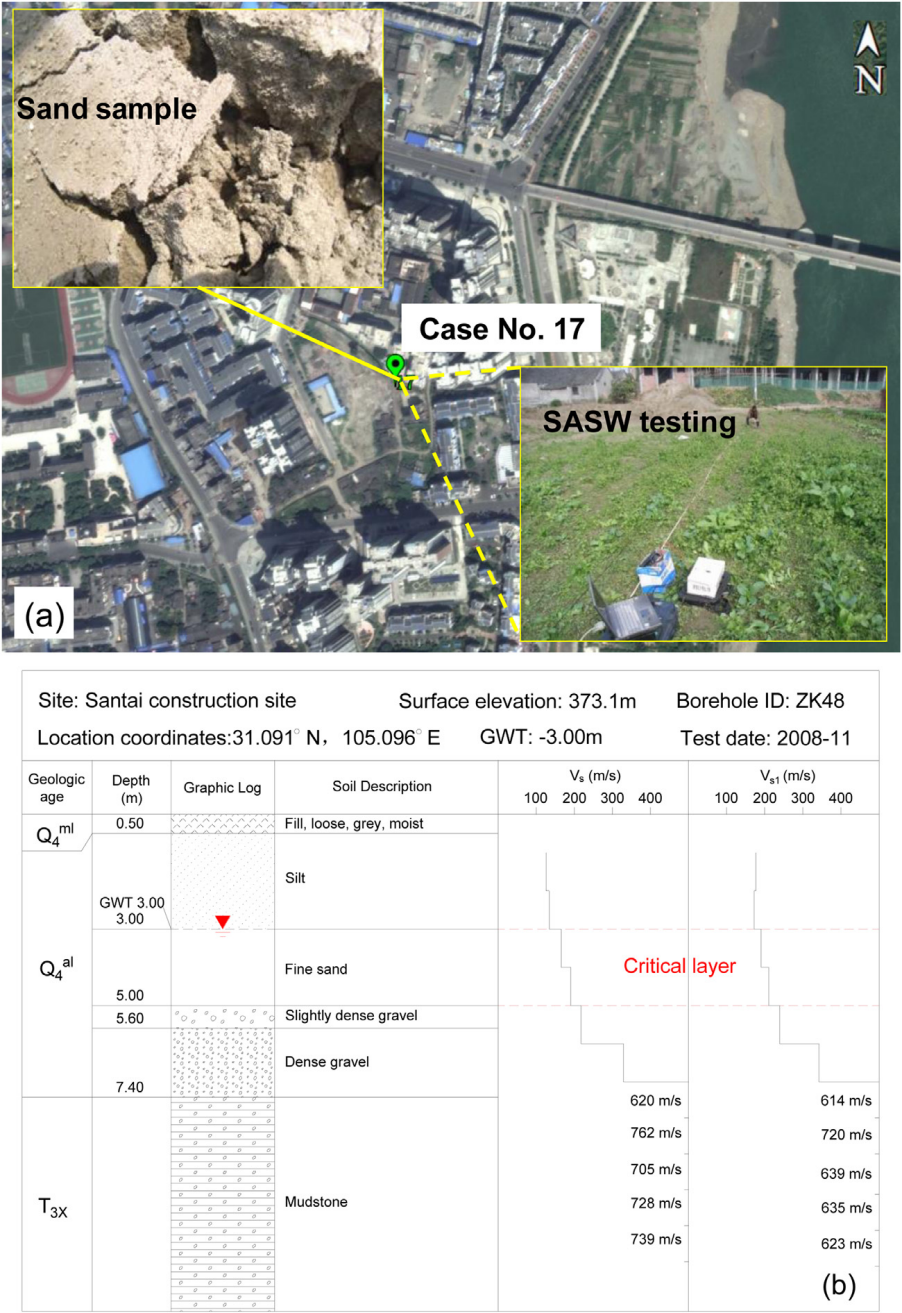
Case No. 17, Santai construction site: (a) site map and field work; (b) soil strata and shear wave velocity profile.

The procedure of *V*_s_-based liquefaction case study is illustrated in Fig. 2. The first task in field liquefaction case study is to select sites within the earthquake affected area, wherein the liquefied and the non-liquefied sites should be equally treated instead of just focusing on the liquefied sites. This can avoid a data bias between the liquefied case histories and the non-liquefied case histories, which is vital to obtain the liquefaction triggering boundary curve for engineering application. The site with features of soil boils or blows, lateral spreading, building tilting or settlement, ground loss and broken lifelines was identified as liquefied, and the liquefaction manifestations were identified by field investigations right after the occurrence of the 12 May 2008 *M*_w_7.9 Wenchuan earthquake. The in-situ soil samples were obtained by core drilling according to Chinese Code for Investigation of Geotechnical Engineering (GB50021-2001, 2009 [4]), and the borehole samples of different depths were taken back to the laboratories to do sieve analysis tests and density tests. By these field investigation and laboratory tests, key parameters including ground water table, soil classification, gravel content, soil density and the soil strata profile could be obtained. At some sites, the in-situ pumping tests were conducted to obtain the permeability coefficient according to reference [4]. The surface inclination angle of each site was determined by dividing the elevation difference by lateral distance (measured by digital laser range finder) of two points at a site. The shear wave velocities of each site were obtained by down-hole testing or spectral analysis of surface wave (SASW) testing after the 2008 Wenchuan earthquake, and at some well-defined sites (e.g., Mianzhu Banqiao School site, Case No.3-5 in Table 1), both SASW and down-hole testings were conducted in parallel to check the reliability of the SASW method by treating the result of down-hole testing as the reference. Typical field work, soil strata and the corresponding profile of shear wave velocity at a site are illustrated in Fig. 3.

The criterion for the identification of the critical layer is to find the soil stratum that is the most likely to trigger and manifest liquefaction at the ground surface for a given site [5]. To specify, the identification of the critical layer should meet the following requirements: (i) the stratum is liquefiable and below the ground water table, this feature could be judged from soil boring and soil classifications via laboratory tests; (ii) the stratum has the lowest normalized shear wave velocity (*V*_s1_), meanwhile the chosen stratum should be as shallow as possible; (iii) one auxiliary identification comes from the surface ejecta which helps to indicate the liquefaction of a specific stratum, but caution should be exercised with gravelly soils as it consists of both sand and gravel particles. As shown in Fig. 3, the critical layer in the soil profile at Santai construction site is identified as the fine sand layer, which has the lowest shear wave velocity and is most likely to trigger and manifest liquefaction at the ground surface. Key parameters of the critical layer at a given site were determined according to the borehole sampling record, field testing of shear wave velocity and laboratory tests for the fine contents, gravel contents and particle size distributions. The mean and variance of the normalized shear wave velocity given in Table 1 could be calculated based on the *V*_s1_ profile within the critical layer as the average thickness-weighted value. The earthquake-induced cyclic stress ratio (CSR) is calculated from PGA estimation in a linear interpolation manner based on the USGS ShakeMap (see Fig. 1(b)–(d)) for the 2008 Wenchuan earthquake. Some key parameters like the normalized shear wave velocity and the CSR could be calculated by using the following equations [6]:

(1) $$V_{s1}=V_{s}C_{V}=V_{s}{\left(\frac{P_{a}}{{\sigma^{\prime }}_{v}}\right)}^{n/2}$$

(2) $$CSR=\frac{\tau_{av}}{{\sigma^{\prime }}_{v}}=0.65(\frac{a_{\max}}{g})(\frac{\sigma_{v}}{{\sigma^{\prime }}_{v}})r_{d}$$

where *P*_a_ is the atmosphere pressure; *n* is the exponent in Hardin equation [7]; *V*_s_ is the shear wave velocity; *a*_max_ is the peak horizontal ground surface acceleration (PGA) in unit of gravity; g is the acceleration of gravity; *σ*_v_ is the total overburden stress at the depth in question; *σ’*_v_ is the effective overburden stress at the depth in question; and *r*_d_ is the shear-mass participation parameter to adjust for the flexibility of the soil profile [8]. The calculation of other parameters such as the standard deviations of these parameters could be found in the related research article [1] or other literatures [9,10].

Based on this procedure, the authors drove through the whole earthquake-impacted areas including the Chengdu plain and the mountain area along the fault rupture, to do reconnaissance work and identify the liquefied and non-liquefied sites from May to early June in 2008, and then carried out field drilling and testing work from July to November for most of the selected sites. Some other sites were investigated later when the site condition allowed doing so. For a few sites where field testing could not be conducted directly, the necessary geotechnical information from the adjacent construction projects are adopted. Finally a total number of 81 well-identified cases are compiled with all detailed information given in the spreadsheet file of raw data, and some key parameters of these cases are summarized in Table 1.

## Declaration of Competing Interest

The authors declare that they have no known competing financial interests or personal relationships which have, or could be perceived to have, influenced the work reported in this article.

## Appendix Supplementary materials

Image, application 1

## References

[1] Zhou Y.G., Xia P., Ling D.S., Chen Y.M. (2020). Liquefaction case studies of gravelly soils during the 2008 Wenchuan earthquake. Eng. Geol..

[2] Cao Z.Z. (2010). Characteristics of soil liquefaction in the great Wenchuan earthquake and procedures for gravelly soil liquefaction evaluation.

[3] Zhao X.Q., Zhao L.J., Wang F.D. (2015). Research status of the quaternary sedimentation in the Chengdu Basin. Sci. Res..

[4] GB50021-2001 (2009). Chinese Code for Investigation of Geotechnical Engineering.

[5] Cubrinovski M., Bray J.D., de la Torre C., Olsen M., Bradley B., Chiaro G., Stocks E., Wotherspoon L., Krall T. (2018). Liquefaction-induced damage and CPT characterization of the reclamations at CentrePort, Wellington. Bull. Seismol. Soc. Am..

[6] Youd T.L., Idriss I.M., Andrus R.D., Arango I., Castro G., Christian J.T., Dobry R., Liam Finn W.D., Harder L.F, Hynes M.E., Ishihara K., Koester J.P., Liao S.S.C., Marcuson III W.F., Martin G.R., Mitchell J.K., Moriwaki Y., Power M.S., Robertson P.K., Seed R.B., Stokoe II K.H. (2001). Liquefaction resistance of soils: summary report from the 1996 NCEER and 1998 NCEER/NSF workshops on evaluation of liquefaction resistance of soils. J. Geotech. Geoenviron. Eng..

[7] Zhou Y.G., Sun Z.B., Chen Y.M. (2018). Zhejiang University benchmark centrifuge test for LEAP-GWU-2015 and liquefaction responses of a sloping ground. Soil Dyn. Earthq. Eng..

[8] Idriss I.M., Boulanger R.W. (2008). Soil Liquefaction During Earthquakes.

[9] Moss R.E.S. (2003). CPT-Based Probabilistic Assessment of Seismic Soil Liquefaction Initiation.

[10] Moss R.E.S. (2008). Quantifying measurement uncertainty of thirty-meter shear-wave velocity. Bull. Seismol. Soc. Am..

